# Supplementary material for: The effect of results-based motivating system on metabolic risk factors of non-communicable diseases: A field trial study
Source: PLoS One. 2024 Oct 17;19(10):e0311507. doi: 10.1371/journal.pone.0311507 (PMC11486381; doi:10.1371/journal.pone.0311507)
Supplement: S2 File — (PDF) [file pone.0311507.s004.pdf]

The impact of a results-based motivating system on population levels of the non-communicable diseases risk factors in Iran: A field trial study

View the latest revision (/trial/774)

History

#	Registration date	Revision Id
3	2020-03-19, 1398/12/29 (/trial/774?revision=129234)	129234
2	2019-05-06, 1398/02/16 (/trial/774?revision=117465)	117465
➜ 1	2018-06-03, 1397/03/13 (/trial/774?revision=47984)	47984

Changes made to previous revision

This is the first revision

Protocol summary

Study aim

Study the impact of a results-based motivating system on population levels of the non-communicable diseases risk factors in Iran: A field trial study

Design

First phase: at this stage, the basic information is collected based on the STEP questionnaire (with biochemical and physical measurements), at which point all four groups will be included in the study. The second phase: by using regular review studies, the best evidence and best practices will be obtained for effective interventions, and then we will train it with health experts and carers to use the methods. Third phase: in this phase, an operational plan with the presence of healthcare professionals and health care staff with the active participation of the research team is based on the initial data provided in the first phase of the study Phase

IV: Using a performance-based incentive system to achieve goals based on initial data. In this regard, the focus group (FGD) method is used to reach the best option for an ideal incentive. In this phase only one group will arrive.

Settings and conduct

This study will be carried out in Bushehr and Semnan universities of medical sciences, Iran.

Participants/Inclusion and exclusion criteria

12 urban health centers and 12 rural health homes

Intervention groups

12 urban health centers and 12 health homes

Main outcome variables

Hypertension; diabetes; insufficient physical activity; tobacco smoking; insufficient intake of fruits/vegetables; body mass index

General information

Reason for update

Acronym

IRPONT

IRCT registration information

IRCT registration number: **IRCT20081205001488N2**
Registration date: **2018-06-03, 1397/03/13**
Registration timing: **registered_while_recruiting**

Last update: **2018-06-03, 1397/03/13**
Update count: **2**

Registration date

2018-06-03, 1397/03/13

Registrant information

Name
Maziar Moradi-Lakeh
Name of organization / entity
Iran University of Medical Sciences
Country
Iran (Islamic Republic of)
Phone
+98 21 8860 2225
Email address
moradilakeh.m@iums.ac.ir

**Recruitment status**	**Recruitment complete**
**Funding source**	National Institute for Medical Research Development (NIMAD)
**Expected recruitment start date**	2018-01-01, 1396/10/11
**Expected recruitment end date**	2020-12-31, 1399/10/11
**Actual recruitment start date**	*empty*
**Actual recruitment end date**	*empty*
**Trial completion date**	*empty*
**Scientific title**	The impact of a results-based motivating system on population levels of the non-communicable diseases risk factors in Iran: A field trial study

Public title

The impact of a results-based motivating system on population levels of the non-communicable diseases risk factors in Iran: A field trial study

Purpose

Health service research

Inclusion/Exclusion criteria

Inclusion criteria:
In the first study, three medical universities will be selected randomly from three different climates, and they will be asked to submit a list of health and medical and health centers in their urban and rural areas, broken down by health centers and health homes. Then the list of networks that have the entry criteria (descriptions in the entry criteria) are prepared and then the selection is made randomly. From each University, four Urban health centers and four health home randomly selected . Four groups (each consisting of a health center and a health center) will be introduced. Universities eligible for entry into the study: 1- Have the consent to cooperate in the study. Cities eligible for entry into the study are: 1. Cities with at least four urban health centers and four health-care homes. Eligible entry centers: 1. Health homes with at least two "Behvarz". 2. City bases Have at least 2 health care staff. And preferably have recruiting staff . 3. health homes that are preferable to the Very small village .eligible staff : Preferably, have fixed forces (recruitment) of health centers and health homes At least 2 years into that center.
Exclusion criteria:

Age

From **30 years** old to **70 years** old

Gender

Both

Phase

N/A

Groups that have been masked

No information

Sample size

Target sample size: **24**

Randomization (investigator's opinion)

Randomized

Randomization description

With a list of networks, health centers and health homes, there will be a simple randomization criterion

Blinding (investigator's opinion)

Not blinded

Blinding description

Placebo

Used

Assignment

Parallel

Other design features

Secondary Ids

empty

Ethics committees

1

Ethics committee

Name of ethics committee
national institute for medical research development

Street address
Tehran, West Fatemi St., Besat Street, No. 21

City
Tehran

Province

Tehran

Postal code

۶۶۹۰۰۹۲۰-۶۶۹۳۸۰۳۷

Approval date

2017-07-31, 1396/05/09

Ethics committee reference number

IR.NIMAD.REC.1396.084

2

Ethics committee

Name of ethics committee

National Institute for Medical Research Development (NIMAD)

Street address

No 21, Besat St, West Fatemi Ave

City

Tehran

Province

Tehran

Postal code

۶۶۹۰۰۹۲۰-۶۶۹۳۸۰۳۷

Approval date

2017-07-31, 1396/05/09

Ethics committee reference number

IR.NIMAD.REC.1396.084

Health conditions studied

1

Description of health condition studied

Risk factors of Non-communicable diseases

**ICD-10 code**	
**ICD-10 code description**	

Primary outcomes

1

**Description**	Population level of uncontrolled hypertension
**Timepoint**	At the beginning of intervention (Month 0), 12 months after starting of intervention, 24 month after starting of intervention
**Method of measurement**	Population Survey

2

**Description**	Population level of Poorly controlled diabetes
**Timepoint**	At the beginning of intervention (Month 0), 12 months after starting of intervention, 24 month after starting of intervention
**Method of measurement**	Population Survey

3

**Description**	Population level of insufficient physical activity
**Timepoint**	At the beginning of intervention (Month 0), 12 months after starting of intervention, 24 month after starting of intervention

**Method of measurement**	Population Survey

4

**Description**	Population level of current tobacco smoking
**Timepoint**	At the beginning of intervention (Month 0), 12 months after starting of intervention, 24 month after starting of intervention
**Method of measurement**	Population Survey

5

**Description**	Population level of insufficient intake of fruits/vegetables
**Timepoint**	At the beginning of intervention (Month 0), 12 months after starting of intervention, 24 month after starting of intervention
**Method of measurement**	Population Survey

6

**Description**	Population level of body mass index
**Timepoint**	At the beginning of intervention (Month 0), 12 months after starting of intervention, 24 month after starting of intervention
**Method of measurement**	Population Survey

Secondary outcomes

empty

Intervention groups

1

Description

Group IV: Assessment of the main NCDs’ risk factors and setting time-bound targets, AND Finding and sharing evidence on effective/efficient interventions for controlling the risk factors AND Operational planning with contribution of local health authoriti

Category

Other

2

Description

Group I: Assessment of the main NCDs’ risk factors and setting time-bound targets.

Category

Other

3

Description

Group II: Assessment of the main NCDs’ risk factors and setting time-bound targets AND Finding and sharing evidence on effective/efficient interventions for controlling the risk factors

Category

Other

4

Description

Group III: Assessment of the main NCDs’ risk factors and setting time-bound targets AND Finding and sharing evidence on effective/efficient interventions for

controlling the risk factors AND Operational planning with contribution of local health authorit

Category

Other

Recruitment centers

1

Recruitment center

Name of recruitment center
Iran university of medical sciences

Full name of responsible person
Maziar Moradi-Lakeh

Street address
Hemat Highway next to Milad Tower, Iran University of Medical Sciences - Faculty of Medicine - Third Floor - Department of Social and Family Medicine

City
Tehran

Province
Tehran

Postal code
۱۴۴۹۶۱۴۵۳۵

Phone
+98 21 8860 2225

Email
mazmoradi@gmail.com

2

Recruitment center

Name of recruitment center
Bushehr University of Medical Sciences

Full name of responsible person
Maziar Moradi-Lakeh

Street address
Bushehr, Sports ST

City
Borazjan

Province
Boushehr

Postal code
۱۴۴۹۶۱۴۵۳۵

Phone
+98 71 3252 2078

Email
mazmoradi@gmail.com

3

Recruitment center

Name of recruitment center
Semnan University of Medical Sciences

Full name of responsible person
Maziar Moradi-Lakeh

Street address
Basij Blvd

City
Damghan

Province
Semnan

Postal code
3519899951

Phone
+98 23 3344 1022

Email
mazmoradi@gmail.com

Sponsors / Funding sources

1

Sponsor

Name of organization / entity
national Institute for Medical Research Development (NIMAD)

Full name of responsible person
Dr. Sayena Rafizadeh - Project #958058

Street address
No 21, Besat St, West Fatemi Ave

City
Tehran

Province
Tehran

Postal code
۱۴۴۹۶۱۴۵۳۵

Phone
+98 21 8860 2225

Email
mazmoradi@gmail.com

**Grant name**	
**Grant code / Reference number**	
**Is the source of funding the same sponsor organization/entity?**	Yes
**Title of funding source**	national Institute for Medical Research Development (NIMAD)
**Proportion provided by this source**	100
**Public or private sector**	Public
**Domestic or foreign origin**	Domestic

Category of foreign source of funding

empty

Country of origin

Type of organization providing the funding

Other

Person responsible for general inquiries

Contact

Name of organization / entity
Preventive Medicine and Public Health Research Center

Full name of responsible person
Maziar Moradi-Lakeh

Position
Professor

Latest degree
Specialist

Other areas of specialty/work
Public Health/Community Medicine

Street address
IUMS, Crossroads of Hemmat-Chamran expressways, Tehran, Iran

City
Tehran

Province
Tehran

Postal code
۱۴۴۹۶۱۴۵۳۵

Phone
+98 21 8860 2225

Fax

Email
mazmoradi@yahoo.com

Web page address

Person responsible for scientific inquiries

Contact

Name of organization / entity
Preventive Medicine and Public Health Research Center

Full name of responsible person
Dr. Maziar Moradi-Lakeh

Position
Professor

Latest degree
Specialist

Other areas of specialty/work
Public Health/Community Medicine

Street address
Iran University of Medical Sciences, Hemmat-Chamran crossroads

City
Tehran

Province
Tehran

Postal code
۱۴۴۹۶۱۴۵۳۵

Phone
+98 21 8860 2225

Fax

Email
mazmoradi@yahoo.com

Web page address

Person responsible for updating data

Contact

Name of organization / entity
Preventive Medicine and Public Health Research Center

Full name of responsible person
Maziar Moradi-Lakeh

Position
Professor

Latest degree
Specialist

Other areas of specialty/work
Public Health/Community Medicine

Street address
IUMS, Crossroads of Hemmat-Chamran expressway

City
Tehran

Province
Tehran

Postal code
1635883813

Phone
+98 21 8860 2225

Fax

Email
mazmoradi@yahoo.com

Web page address

Sharing plan

**Deidentified Individual Participant Data Set (IPD)**	Yes - There is a plan to make this available
**Study Protocol**	Yes - There is a plan to make this available
**Statistical Analysis Plan**	Yes - There is a plan to make this available
**Informed Consent Form**	Yes - There is a plan to make this available
**Clinical Study Report**	

Yes - There is a plan to make this available

**Analytic Code**	Yes - There is a plan to make this available
**Data Dictionary**	Yes - There is a plan to make this available
**Title and more details about the data/document**	Access to relevant files is possible one year after the publication, with correspondence email: mazmoradi@gmail.com.
**When the data will become available and for how long**	One year after publication
**To whom data/document is available**	Academic researchers
**Under which criteria data/document could be used**	There is no limitation to the analysis
**From where data/document is obtainable**	Contact with email: mazmoradi@gmail.com
**What processes are involved for a request to access data/document**	Study aims of the study, about 10 to 21 days.
**Comments**	

- Help (/)

Tel:
Working hours:
8:00 - 15:30 Tehran time
11:30 - 19:00 GMT

0098 21 8670 5503

During COVID-19 Epidemic at working times:
0098 936 770 7834

Fax:
0098 21 8670 5503

Email:
admin@irct.ir (mailto:admin@irct.ir)

Directly contacting the manager:
0098 912 778 2686

Address:
IRCT administration team,
Central Library Building, Iran University Campus,
Hemmat freeway, next to Milad tower,
Tehran, 14496-14535
Iran

Copyright © IRCT 2008-2023. All Rights Reserved.
