# Supplementary material for: The effect of results-based motivating system on metabolic risk factors of non-communicable diseases: A field trial study
Source: PLoS One. 2024 Oct 17;19(10):e0311507. doi: 10.1371/journal.pone.0311507 (PMC11486381; doi:10.1371/journal.pone.0311507)
Supplement: S3 File — (PDF) [file pone.0311507.s005.pdf]

The impact of a results-based motivating system on population levels of the non-communicable diseases risk factors in Iran: A field trial study

View the latest revision (/trial/774)

History

#	Registration date	Revision Id
3	2020-03-19, 1398/12/29 (/trial/774?revision=129234)	129234
➜ 2	2019-05-06, 1398/02/16 (/trial/774?revision=117465)	117465
1	2018-06-03, 1397/03/13 (/trial/774?revision=47984)	47984

Changes made to previous revision

Help:

Red color represents old content. It may be empty showing addition in the new version.
Green color represents new content. It may be empty showing deletion in the new version.

Inline	Side by side
Added new contents, ~~deleted old contents,~~ contents that are not changed.	Added new contents, contents that are not changed.<br>Deleted old contents, contents that are not changed.

Inline example table:

New table contents	New table contents
~~Old table contents~~	~~Old table contents~~
Unchanged contents	Unchanged contents

Side by side example tables:

Old table contents	Old table contents
Unchanged contents	Unchanged contents

New table contents	New table contents
Unchanged contents	Unchanged contents

Protocol summary

Inline | Side by side

Design - English

First phase: at this stage, the basic information is collected based on the STEP questionnaire (with biochemical and physical measurements), at which point all four groups will be included in the study. The second phase: by using regular review studies, the best evidence and best practices will be obtained for effective interventions, and then we will train it with health experts and carers to use the methods. Third phase: in this phase, an operational plan with the presence of healthcare professionals and health care staff with the active participation of the research team is based on the initial data provided in the first phase of the study Phase IV: Using a performance-based incentive system to achieve goals based on initial data. In this regard, the focus group (FGD) method is used to reach the best option for an ideal incentive. In this phase only one group will arrive.

First phase: at this stage, the basic information is collected based on the STEP questionnaire (with biochemical and physical measurements), at which point all four groups will be included in the study. The second phase: by using regular review studies, the best evidence and best practices will be obtained for effective interventions, and then we will train it with health experts and carers to use the methods. Third phase: in this phase, an operational plan with the presence of healthcare professionals and health care staff with the active participation of the research team is based on the initial data provided in the first phase of the study Phase IV: Using a performance-based incentive system to achieve goals based on initial data. In this regard, the focus group (FGD) method is used to reach the best option for an ideal incentive. In this phase only one group will arrive. * It should be noted that 4 rural centers and 4 urban centers will not receive any intervention.

Design - Persian

فاز اول: در این مرحله اطلاعات پایه بر اساس پرسشنامه STEP و جمع آوری میگردد (همراه با سنجش های بیوشیمیایی و جسمی) ، در این مرحله هر چهار گروه وارد مطالعه خواهند شد. فاز دوم: با استفاده از مطالعات مروری منظم، بهترین شواهد و بهترین شیوه ها در جهت مداخلات اثر بخش بدست خواهد آمد و سپس آن را به کارشناسان و مراقبین سلامت آموزش خواهیم داد فاز سوم: در این فاز، برنامه عملیاتی با حضور کارشناسان و کارکنان مراقب سلامت با مشارکت فعال تیم پژوهشی بر اساس داده های اولیه مطالعه که در فاز اول تهیه شده است تنظیم می گردد فاز چهارم: استفاده از سیستم تشویق مبتنی بر عملکرد درخصوص رسیدن به اهدافی که بر اساس داده های اولیه تنظیم گردیده است. در این راستا برای رسیدن به بهترین گزینه جهت یک مشوق ایده آل از روش focus group(FGD) استفاده می گردد. در این فاز تنها یک گروه وارد خواهد شد.

فاز اول: در این مرحله اطلاعات پایه بر اساس پرسشنامه STEP و جمع آوری میگردد (همراه با سنجش های بیوشیمیایی و جسمی) ، در این مرحله هر چهار گروه وارد مطالعه خواهند شد. فاز دوم: با استفاده از مطالعات مروری منظم، بهترین شواهد و بهترین شیوه ها در جهت مداخلات اثر بخش بدست خواهد آمد و سپس آن را به کارشناسان و مراقبین سلامت آموزش خواهیم داد فاز سوم: در این فاز، برنامه عملیاتی با حضور کارشناسان و کارکنان مراقب سلامت با مشارکت فعال تیم پژوهشی بر اساس داده های اولیه مطالعه که در فاز اول تهیه شده است تنظیم می گردد فاز چهارم: استفاده از سیستم تشویق مبتنی بر عملکرد درخصوص رسیدن به اهدافی که بر اساس داده های اولیه تنظیم گردیده است. در این راستا برای رسیدن به بهترین گزینه جهت یک مشوق ایده آل از روش focus group(FGD) استفاده می گردد. در این فاز تنها یک گروه وارد خواهد شد. *البته قابل ذکر است که 4 مرکز روستایی و 4 مرکز شهری هیچگونه مداخله ای دریافت نخواهند کرد.

Settings and conduct - English

This study will be carried out in Bushehr and Semnan universities of medical sciences, Iran.

This study will be carried out in Bushehr (Dashtestan), Semnan (Damqan and Garmsar) and Iran (shahryar) universities of medical sciences, .

Settings and conduct - Persian

این مطالعه در دانشگاههای علوم پزشکی ایران، بوشهر و سمنان انجام خواهد گرفت

این مطالعه در دانشگاههای علوم پزشکی ایران(شهریار)، بوشهر (دشتستان)و سمنان(دامغان و گرمسار) انجام خواهد گرفت

Participants/Inclusion and exclusion criteria - English

12 urban health centers and 12 rural health homes

16 urban health centers and 16 rural health homes

Participants/Inclusion and exclusion criteria - Persian

12 پایگاه بهداشتی شهری و 12 خانه بهداشت

16 پایگاه بهداشتی شهری و 16 خانه بهداشت

Intervention groups - English

12 urban health centers and 12 health homes

16 urban health centers and 16 health homes

Intervention groups - Persian

12 پایگاه بهداشتی شهری و 12 خانه بهداشت

16 پایگاه بهداشتی شهری و 16 خانه بهداشت

General information

Inline | Side by side

Participant

Care provider

Investigator

Outcome assessor

Data analyser

Data and Safety Monitoring Board

Target sample size

24

32

More than 1 sample in each individual

No

Yes

Number of samples in each individual

empty

40

Reason for update - English

empty

Selection of other clusters to reduce potential data contamination

Reason for update - Persian

empty

انتخاب خوشه های دیگر برای کاهش وجود آلودگی احتمالی داده ها

Inclusion criteria - English

In the first study, three medical universities will be selected randomly from three different climates, and they will be asked to submit a list of health and medical and health centers in their urban and rural areas, broken down by health centers and health homes. Then the list of networks that have the entry criteria (descriptions in the entry criteria) are prepared and then the selection is made randomly. From each University, four Urban health centers and four health home randomly selected . Four groups (each consisting of a health center and a health center) will be introduced.
Universities eligible for entry into the study: 1- Have the consent to cooperate in the study. Cities eligible for entry into the study are: 1. Cities with at least four urban health centers and four health-care homes. Eligible entry centers: 1. Health homes with at least two "Behvarz". 2. City bases Have at least 2 health care staff. And preferably have recruiting staff . 3. health homes that are preferable to the Very small village .eligible staff : Preferably, have fixed forces (recruitment) of health centers and health homes At least 2 years into that center.

In the first study, three medical universities will be selected randomly from three different climates, and they will be asked to submit a list of health and medical and health centers in their urban and rural areas, broken down by health centers and health homes. Then the list of networks that have the entry criteria (descriptions in the entry criteria) are prepared and then the selection is made randomly. From each University, four Urban health centers and four health home randomly selected . Four groups (each consisting of a health center and a health center) will be introduced.Of course, to control the likelihood of data contamination, one of the universities(Semnan), in addition to the intervention cluster city, will select another city(Garmsar) with 4 urban clusters and 4 rural clusters, which will receive no intervention.
Universities eligible for entry into the study: 1- Have the consent to cooperate in the study. Cities eligible for entry into the study are: 1. Cities with at least four urban health centers and four health-care homes. Eligible entry centers: 1. Health homes with at least two "Behvarz". 2. City bases Have at least 2 health care staff. And preferably have recruiting staff . 3. health homes that are preferable to the Very small village .eligible staff : Preferably, have fixed forces (recruitment) of health centers and health homes At least 2 years into that center.

Inclusion criteria - Persian

در این مطالعه در ابتدا سه دانشگاه علوم پزشکی از سه اقلیم متفاوت بصورت غیرتصادفی انتخاب خواهند شد و از آنها درخواست می گردد تا لیست شبکه های بهداشت و درمان و مراکز بهداشتی شهری و روستایی خود را به تفکیک پایگاههای بهداشتی و خانه های بهداشت ارسال نمایند. سپس لیست شبکه هایی که دارای معیار ورود(توضیحات در قسمت معیارهای ورود) هستند تهیه گردیده وپس از آن انتخاب بصورت تصادفی صورت می پذیرد. از هر دانشگاه 4 پایگاه بهداشتی شهری و 4 خانه بهداشت که بصورت تصادفی انتخاب می گردند، مورد مطالعه قرار خواهد گرفت(ترجیحاً از یک شهرستان باشد) 4 گروه (هر گروه متشکل از یک پایگاه بهداشتی و یک خانه بهداشت می باشد) معرفی می گردد.
دانشگاههای واجد شرایط ورود به مطالعه:1- داشتن رضایت جهت همکاری در طرح شهرستان های واجد شرایط ورود به مطالعه:1- شهرستان هایی که حداقل دارای 4 پایگاه بهداشتی شهری و 4 خانه بهداشت باشد.مراکز واجد شرایط ورود به مطالعه:1- خانه های بهداشتی که حداقل دارای دو نیروی بهورز باشند.2- پایگاههای شهری که حداقل دارای 2 نیروی مراقب سلامت باشد. و ترجیحاً دارای نیروهای ثابت استخدامی(غیر طرح نیروی انسانی) باشند.3- خانه بهداشت هایی که ترجیحا روستای قمر نداشته باشند.نیروهای ارائه دهنده خدمت واجد شرایط ورود به مطالعه:ترجیحاً از نیروهایی ثابت(استخدامی) پایگاههای بهداشتی و خانه بهداشت باشند که حداقل تا 2 سال آینده در آن مرکز مستقر باشند.

در این مطالعه در ابتدا سه دانشگاه علوم پزشکی (ایران، بوشهر و سمنان) از سه اقلیم متفاوت بصورت غیرتصادفی انتخاب خواهند شد و از آنها درخواست می گردد تا لیست شبکه های بهداشت و درمان و مراکز بهداشتی شهری و روستایی خود را به تفکیک پایگاههای بهداشتی و خانه های بهداشت ارسال نمایند. سپس لیست شبکه هایی که دارای معیار ورود(توضیحات در قسمت معیارهای ورود) هستند تهیه گردیده وپس از آن انتخاب بصورت تصادفی صورت می پذیرد. از

هر دانشگاه سه شهرستان (شهریار، دشتستان و دامغان) و از هر شهرستان 4 پایگاه بهداشتی شهری و 4 خانه بهداشت که بصورت تصادفی انتخاب می گردند، مورد مطالعه قرار خواهد گرفت(ترجیحاً از یک یا دو شهرستان باشد) 4 گروه (هر گروه متشکل از یک پایگاه بهداشتی و یک خانه بهداشت می باشد) معرفی می گردد.البته برای کنترل احتمال آلودگی داده ها، از یکی از دانشگاهها (سمنان)، علاوه بر شهرستان دارای خوشه های مداخله، یک شهرستان دیگر (گرمسار)با 4 خوشه شهری و 4 خوشه روستایی انتخاب خواهد شد، که هیچ گونه مداخله ای را دریافت نخواهد کرد.
دانشگاههای واجد شرایط ورود به مطالعه:1- داشتن رضایت جهت همکاری در طرح شهرستان های واجد شرایط ورود به مطالعه:1- شهرستان هایی که حداقل دارای 4 پایگاه بهداشتی شهری و 4 خانه بهداشت باشد.مراکز واجد شرایط ورود به مطالعه:1- خانه های بهداشتی که حداقل دارای دو نیروی بهورز باشند.2- پایگاههای شهری که حداقل دارای 2 نیروی مراقب سلامت باشد. و ترجیحاً دارای نیروهای ثابت استخدامی(غیر طرح نیروی انسانی) باشند.3- خانه بهداشت هایی که ترجیحا روستای قمر نداشته باشند.نیروهای ارائه دهنده خدمت واجد شرایط ورود به مطالعه:ترجیحاً از نیروهایی ثابت(استخدامی) پایگاههای بهداشتی و خانه بهداشت باشند که حداقل تا 2 سال آینده در آن مرکز مستقر باشند.

Exclusion criteria - English

Disagreement with the text of the memorandum, by the relevant authorities of the universities

Exclusion criteria - Persian

با در دست داشتن

عدم موافقت با متن تفاهم نامه، توسط مسئولین ذیربط دانشگاهها

Other design features - English

This is a field trial. Interventions are performed at the first health level of the selected areas, and the final outcome is measured using population surveys.

Other design features - Persian

این یک کارازمایی عرصه است. مداخلات در سطح سانه بهداشتی عرصه های منتخب انجام می گیرد و پیامد نهایی با استفاده از پیمایش جمعیتی سنجیده می شود.

این یک کارازمایی عرصه است. مداخلات در سطح اول بهداشتی عرصه های منتخب انجام می گیرد و پیامد نهایی با استفاده از پیمایش جمعیتی سنجیده می شود.

Blinding description - English

empty

Blinding description - Persian

empty

Description of samples in each individual - English

24 fileds

Thirty-two clusters are considered and for each cluster, 40 samples (individuals with inclusion criteria) will be provided.

Description of samples in each individual - Persian

عرصه تحقیقاتی 24

تعداد 32 خوشه مد نظر می باشد و در هر خوشه، 40 نمونه (نفر که دارای معیار ورود می باشند) تهیه خواهد شد

Intervention groups

Inline | Side by side

#1

Category

empty

Other

Description - English

empty

Control group: A county (Garmsar County) consisting of 4 health houses and 4 health center will not receive any intervention, and only three survey will be conducted.

Description - Persian

empty

گروه کنترل: یک شهرستان(شهرستان گرمسار) متشکل از 4 خانه بهداشت و 4 پایگاه بهداشتی شهری هیچگونه مداخله ای را دریافت نخواهند کرد و تنها سه مرتبه پرسشگری به همراه تن سنجی و نمونه گیری خون انجام خواهد شد

Recruitment centers

Inline | Side by side

#1

Recruitment center

Name of recruitment center - English: Iran university of medical sciences
Name of recruitment center - Persian: دانشگاه علوم پزشکی ایران
Full name of responsible person - English: Maziar Moradi-Lakeh
Full name of responsible person - Persian: مازیار مرادی لاکه
Street address - English: Hemat Highway next to Milad Tower, Iran University of Medical Sciences - Faculty of Medicine - Third Floor - Department of Social and Family Medicine
Street address - Persian: بزرگراه همت جنب برج میلاد، دانشگاه علوم پزشکی ایران - دانشکده پزشکی- طبقه سوم -

گروه پزشکی اجتماعی و خانواده
City - English: Tehran
City - Persian: تهران
Province: Tehran
Country: Iran (Islamic Republic of)
Postal code: ۱۴۴۹۶۱۴۵۳۵
Phone: +98 21 8860 2225
Fax:
Email: mazmoradi@gmail.com
Web page address:

Name of recruitment center - English: Iran university of medical sciences
Name of recruitment center - Persian: دانشگاه علوم پزشکی ایران
Full name of responsible person - English: Maziar Moradi-Lakeh
Full name of responsible person - Persian: مازیار مرادی لاکه
Street address - English: Shahriar Health Network
Street address - Persian: شبکه بهداشت و درمان شهرستان شهریار
City - English: Shahriar
City - Persian: شهریار
Province: Tehran
Country: Iran (Islamic Republic of)
Postal code: ۱۴۴۹۶۱۴۵۳۵
Phone: +98 21 8860 2225
Fax:
Email: mazmoradi@gmail.com
Web page address:

#2

Recruitment center

Name of recruitment center - English: Semnan University of Medical Sciences
Name of recruitment center - Persian: دانشگاه علوم پزشکی سمنان
Full name of responsible person - English: Maziar Moradi-Lakeh
Full name of responsible person - Persian: مازیار مرادی لاکه
Street address - English: Shahid Rajaie Street
Street address - Persian: خیابان شهید رجایی
City - English: Garmsar
City - Persian: گرمسار
Province: Semnan
Country: Iran (Islamic Republic of)
Postal code: 3519899951
Phone: +98 23 3422 8830
Fax:
Email: mazmoradi@gmail.com
Web page address:

Protocol summary

Study aim

Study the impact of a results-based motivating system on population levels of the non-communicable diseases risk factors in Iran: A field trial study

Design

First phase: at this stage, the basic information is collected based on the STEP questionnaire (with biochemical and physical measurements), at which point all four groups will be included in the study. The second phase: by using regular review studies, the best evidence and best practices will be obtained for effective interventions, and then we will train it with health experts and carers to use the methods. Third phase: in this phase, an operational plan with the presence of healthcare professionals and health care staff with the active participation of the research team is based on the initial data provided in the first phase of the study Phase IV: Using a performance-based incentive system to achieve goals based on initial data. In this regard, the focus group (FGD) method is used to reach the best option for an ideal incentive. In this phase only one group will arrive. * It should be noted that 4 rural centers and 4 urban centers will not receive any intervention.

Settings and conduct

This study will be carried out in Bushehr (Dashtestan), Semnan (Damqan and Garmsar) and Iran (shahryar) universities of medical sciences, .

Participants/Inclusion and exclusion criteria

16 urban health centers and 16 rural health homes

Intervention groups

16 urban health centers and 16 health homes

Main outcome variables

Hypertension; diabetes; insufficient physical activity; tobacco smoking; insufficient intake of fruits/vegetables; body mass index

General information

Reason for update

Selection of other clusters to reduce potential data contamination

Acronym

IRPONT

IRCT registration information

IRCT registration number: **IRCT20081205001488N2**
Registration date: **2018-06-03, 1397/03/13**
Registration timing: **registered_while_recruiting**

Last update: **2020-01-10, 1398/10/20**
Update count: **2**

Registration date

2018-06-03, 1397/03/13

Registrant information

Name
Maziar Moradi-Lakeh
Name of organization / entity
Iran University of Medical Sciences
Country
Iran (Islamic Republic of)
Phone
+98 21 8860 2225
Email address
moradilakeh.m@iums.ac.ir

Recruitment status

Recruitment complete

Funding source

National Institute for Medical Research Development (NIMAD)

Expected recruitment start date

2018-01-01, 1396/10/11

Expected recruitment end date

2020-12-31, 1399/10/11

Actual recruitment start date

empty

Actual recruitment end date

empty

Trial completion date

empty

Scientific title

The impact of a results-based motivating system on population levels of the non-communicable diseases risk factors in Iran: A field trial study

Public title

The impact of a results-based motivating system on population levels of the non-communicable diseases risk factors in Iran: A field trial study

Purpose

Health service research

Inclusion/Exclusion criteria

Inclusion criteria:
In the first study, three medical universities will be selected randomly from three different climates, and they will be asked to submit a list of health and medical and health centers in their urban and rural areas, broken down by health centers and health homes. Then the list of networks that have the entry criteria (descriptions in the entry criteria) are prepared and then the selection is made randomly. From each University, four Urban health centers and four health home randomly selected . Four groups (each consisting of a health center and a health center) will be introduced.Of course, to control the likelihood of data contamination, one of the universities(Semnan), in addition to the intervention cluster city, will select another city(Garmsar) with 4

urban clusters and 4 rural clusters, which will receive no intervention. Universities eligible for entry into the study: 1- Have the consent to cooperate in the study. Cities eligible for entry into the study are: 1. Cities with at least four urban health centers and four health-care homes. Eligible entry centers: 1. Health homes with at least two "Behvarz". 2. City bases Have at least 2 health care staff. And preferably have recruiting staff . 3. health homes that are preferable to the Very small village .eligible staff : Preferably, have fixed forces (recruitment) of health centers and health homes At least 2 years into that center.
Exclusion criteria:
Disagreement with the text of the memorandum, by the relevant authorities of the universities

Age

From **30 years** old to **70 years** old

Gender

Both

Phase

N/A

Groups that have been masked

No information

Sample size

Target sample size: **32**
More than 1 sample in each individual
Number of samples in each individual: **40**
Thirty-two clusters are considered and for each cluster, 40 samples (individuals with inclusion criteria) will be provided.

Randomization (investigator's opinion)

Randomized

Randomization description

With a list of networks, health centers and health homes, there will be a simple randomization criterion

Blinding (investigator's opinion)

Not blinded

Blinding description

Placebo

Used

Assignment

Parallel

Other design features

This is a field trial. Interventions are performed at the first health level of the selected areas, and the final outcome is measured using population surveys.

Secondary Ids

empty

Ethics committees

1

Ethics committee

Name of ethics committee
national institute for medical research development

Street address
Tehran, West Fatemi St., Besat Street, No. 21

City
Tehran

Province
Tehran

Postal code
۶۶۹۰۰۹۲۰-۶۶۹۳۸۰۳۷

Approval date

2017-07-31, 1396/05/09

Ethics committee reference number

IR.NIMAD.REC.1396.084

2

Ethics committee

Name of ethics committee
National Institute for Medical Research Development (NIMAD)

Street address
No 21, Besat St, West Fatemi Ave

City
Tehran

Province
Tehran

Postal code
۶۶۹۰۰۹۲۰-۶۶۹۳۸۰۳۷

Approval date

2017-07-31, 1396/05/09

Ethics committee reference number

IR.NIMAD.REC.1396.084

Health conditions studied

1

Description of health condition studied

Risk factors of Non-communicable diseases

ICD-10 code

ICD-10 code description

Primary outcomes

1

Description

Population level of uncontrolled hypertension

Timepoint

At the beginning of intervention (Month 0), 12 months after starting of intervention, 24 month after starting of intervention

Method of measurement

Population Survey

2

Description

Population level of Poorly controlled diabetes

Timepoint

At the beginning of intervention (Month 0), 12 months after starting of intervention, 24 month after starting of intervention

Method of measurement

Population Survey

3

Description

Population level of insufficient physical activity

Timepoint

At the beginning of intervention (Month 0), 12 months after starting of intervention, 24 month after starting of intervention

Method of measurement

Population Survey

4

Description

Population level of current tobacco smoking

**Timepoint**	At the beginning of intervention (Month 0), 12 months after starting of intervention, 24 month after starting of intervention
**Method of measurement**	Population Survey

5

**Description**	Population level of insufficient intake of fruits/vegetables
**Timepoint**	At the beginning of intervention (Month 0), 12 months after starting of intervention, 24 month after starting of intervention
**Method of measurement**	Population Survey

6

**Description**	Population level of body mass index
**Timepoint**	At the beginning of intervention (Month 0), 12 months after starting of intervention, 24 month after starting of intervention
**Method of measurement**	Population Survey

Secondary outcomes

empty

Intervention groups

1

**Description**	

	Group IV: Assessment of the main NCDs' risk factors and setting time-bound targets, AND Finding and sharing evidence on effective/efficient interventions for controlling the risk factors AND Operational planning with contribution of local health authoriti
**Category**	Other
2	
**Description**	Group I: Assessment of the main NCDs' risk factors and setting time-bound targets.
**Category**	Other
3	
**Description**	Group II: Assessment of the main NCDs' risk factors and setting time-bound targets AND Finding and sharing evidence on effective/efficient interventions for controlling the risk factors
**Category**	Other
4	
**Description**	Group III: Assessment of the main NCDs' risk factors and setting time-bound targets AND Finding and sharing evidence on effective/efficient interventions for controlling the risk factors AND Operational planning with contribution of local health authorit
**Category**	Other
5	
**Description**	

Control group: A county (Garmsar County) consisting of 4 health houses and 4 health center will not receive any intervention, and only three survey will be conducted.

Category

Other

Recruitment centers

1

Recruitment center

Name of recruitment center
Iran university of medical sciences

Full name of responsible person
Maziar Moradi-Lakeh

Street address
Shahriar Health Network

City
Shahriar

Province
Tehran

Postal code
۱۴۴۹۶۱۴۵۳۵

Phone
+98 21 8860 2225

Email
mazmoradi@gmail.com

2

Recruitment center

Name of recruitment center
Bushehr University of Medical Sciences

Full name of responsible person
Maziar Moradi-Lakeh

Street address
Bushehr, Sports ST

City
Borazjan

Province
Boushehr

Postal code
۱۴۴۹۶۱۴۵۳۵

Phone
+98 71 3252 2078

Email
mazmoradi@gmail.com

3

Recruitment center

Name of recruitment center
Semnan University of Medical Sciences

Full name of responsible person
Maziar Moradi-Lakeh

Street address
Basij Blvd

City
Damghan

Province
Semnan

Postal code
3519899951

Phone
+98 23 3344 1022

Email
mazmoradi@gmail.com

4

Recruitment center

Name of recruitment center
Semnan University of Medical Sciences

Full name of responsible person

Maziar Moradi-Lakeh

Street address
Shahid Rajaie Street

City
Garmsar

Province
Semnan

Postal code
3519899951

Phone
+98 23 3422 8830

Email
mazmoradi@gmail.com

Sponsors / Funding sources

1

Sponsor

Name of organization / entity
national Institute for Medical Research Development (NIMAD)

Full name of responsible person
Dr. Sayena Rafizadeh - Project #958058

Street address
No 21, Besat St, West Fatemi Ave

City
Tehran

Province
Tehran

Postal code
۱۴۴۹۶۱۴۵۳۵

Phone
+98 21 8860 2225

Email
mazmoradi@gmail.com

Grant name

**Grant code / Reference number**	
**Is the source of funding the same sponsor organization/entity?**	Yes
**Title of funding source**	national Institute for Medical Research Development (NIMAD)
**Proportion provided by this source**	100
**Public or private sector**	Public
**Domestic or foreign origin**	Domestic
**Category of foreign source of funding**	*empty*
**Country of origin**	
**Type of organization providing the funding**	Other

Person responsible for general inquiries

Contact

Name of organization / entity
Preventive Medicine and Public Health Research Center

Full name of responsible person
Maziar Moradi-Lakeh

Position
Professor

Latest degree
Specialist

Other areas of specialty/work
Public Health/Community Medicine

Street address
IUMS, Crossroads of Hemmat-Chamran expressways, Tehran, Iran

City
Tehran

Province
Tehran

Postal code
۱۴۴۹۶۱۴۵۳۵

Phone
+98 21 8860 2225

Fax

Email
mazmoradi@yahoo.com

Web page address

Person responsible for scientific inquiries

Contact

Name of organization / entity
Preventive Medicine and Public Health Research Center

Full name of responsible person
Dr. Maziar Moradi-Lakeh

Position
Professor

Latest degree
Specialist

Other areas of specialty/work
Public Health/Community Medicine

Street address
Iran University of Medical Sciences, Hemmat-Chamran crossroads

City

Tehran

Province
Tehran

Postal code
۱۴۴۹۶۱۴۵۳۵

Phone
+98 21 8860 2225

Fax

Email
mazmoradi@yahoo.com

Web page address

Person responsible for updating data

Contact

Name of organization / entity
Preventive Medicine and Public Health Research Center

Full name of responsible person
Maziar Moradi-Lakeh

Position
Professor

Latest degree
Specialist

Other areas of specialty/work
Public Health/Community Medicine

Street address
IUMS, Crossroads of Hemmat-Chamran expressway

City
Tehran

Province
Tehran

Postal code
1635883813

Phone
+98 21 8860 2225

Fax

Email
mazmoradi@yahoo.com
Web page address

Sharing plan

**Deidentified Individual Participant Data Set (IPD)**	Yes - There is a plan to make this available
**Study Protocol**	Yes - There is a plan to make this available
**Statistical Analysis Plan**	Yes - There is a plan to make this available
**Informed Consent Form**	Yes - There is a plan to make this available
**Clinical Study Report**	Yes - There is a plan to make this available
**Analytic Code**	Yes - There is a plan to make this available
**Data Dictionary**	Yes - There is a plan to make this available
**Title and more details about the data/document**	Access to relevant files is possible one year after the publication, with correspondence email: mazmoradi@gmail.com.
**When the data will become available and for how long**	One year after publication
**To whom data/document is available**	Academic researchers

**Under which criteria data/document could be used**	
	There is no limitation to the analysis
**From where data/document is obtainable**	
	Contact with email: mazmoradi@gmail.com
**What processes are involved for a request to access data/document**	
	Study aims of the study, about 10 to 21 days.
**Comments**	

Tel:
Working hours:
8:00 - 15:30 Tehran time
11:30 - 19:00 GMT

0098 21 8670 5503

During COVID-19 Epidemic at working times:
0098 936 770 7834

Fax:
0098 21 8670 5503

Email:
admin@irct.ir (mailto:admin@irct.ir)

Directly contacting the manager:
0098 912 778 2686

Address:
IRCT administration team,
Central Library Building, Iran University Campus,
Hemmat freeway, next to Milad tower,
Tehran, 14496-14535
Iran

Copyright © IRCT 2008-2023. All Rights Reserved.
