# Supplementary material for: The effect of results-based motivating system on metabolic risk factors of non-communicable diseases: A field trial study
Source: PLoS One. 2024 Oct 17;19(10):e0311507. doi: 10.1371/journal.pone.0311507 (PMC11486381; doi:10.1371/journal.pone.0311507)
Supplement: S4 File — (PDF) [file pone.0311507.s006.pdf]

# Clinical Trial Protocol
# Iranian Registry of Clinical Trials

23 Jul 2023

## The impact of a results-based motivating system on population levels of the non-communicable diseases risk factors in Iran: A field trial study

### Protocol summary

**Study aim**

Study the impact of a results-based motivating system on population levels of the non-communicable diseases risk factors in Iran: A field trial study

**Design**

First phase: at this stage, the basic information is collected based on the STEP questionnaire (with biochemical and physical measurements), at which point all four groups will be included in the study. The second phase: by using regular review studies, the best evidence and best practices will be obtained for effective interventions, and then we will train it with health experts and carers to use the methods. Third phase: in this phase, an operational plan with the presence of healthcare professionals and health care staff with the active participation of the research team is based on the initial data provided in the first phase of the study Phase IV:  Using a performance-based incentive system to achieve goals based on initial data. In this regard, the focus group (FGD) method is used to reach the best option for an ideal incentive. In this phase only one group will arrive. * It should be noted that 4 rural centers and 4 urban centers will not receive any intervention.

**Settings and conduct**

This study will be carried out in Bushehr (Dashtestan), Semnan (Damqan and Garmsar) and Iran (shahryar) universities of medical sciences, .

**Participants/Inclusion and exclusion criteria**

16 urban health centers and 16 rural health homes

**Intervention groups**

16 urban health centers and 16 health homes

**Main outcome variables**

Hypertension; diabetes; insufficient physical activity; tobacco smoking; insufficient intake of fruits/vegetables; body mass index

### General information

**Reason for update**

revised based on reviewer comment (of course, it was done in consultation with an epidemiologist)

**Acronym**

IRPONT

**IRCT registration information**

IRCT registration number: **IRCT20081205001488N2**
Registration date: **2018-06-03, 1397/03/13**
Registration timing: **prospective**

Last update: **2020-04-12, 1399/01/24**
Update count: **2**

**Registration date**

2018-06-03, 1397/03/13


**Registrant information**

**Name**

Maziar Moradi-Lakeh

**Name of organization / entity**

Iran University of Medical Sciences

**Country**

Iran (Islamic Republic of)

**Phone**

+98 21 8860 2225

**Email address**

moradilakeh.m@iums.ac.ir


**Recruitment status**

**Recruitment complete**


**Funding source**

National Institute for Medical Research Development (NIMAD)


**Expected recruitment start date**

2018-06-17, 1397/03/27

**Expected recruitment end date**

2021-03-18, 1399/12/28

**Actual recruitment start date**

empty

**Actual recruitment end date**

empty

**Trial completion date**

empty

**Scientific title**
The impact of a results-based motivating system on population levels of the non-communicable diseases risk factors in Iran: A field trial study

**Public title**
The impact of a results-based motivating system on population levels of the non-communicable diseases risk factors in Iran: A field trial study

**Purpose**
Health service research

**Inclusion/Exclusion criteria**
**Inclusion criteria:**
In the first study, three medical universities will be selected randomly from three different climates, and they will be asked to submit a list of health and medical and health centers in their urban and rural areas, broken down by health centers and health homes. Then the list of networks that have the entry criteria (descriptions in the entry criteria) are prepared and then the selection is made randomly. From each University, four Urban health centers and four health home randomly selected . Four groups (each consisting of a health center and a health center) will be introduced.Of course, to control the likelihood of data contamination, one of the universities(Ŝemnan), in addition to the intervention cluster city, will select another city(Garmsar) with 4 urban clusters and 4 rural clusters, which will receive no intervention. Universities eligible for entry into the study: 1. Have the consent to cooperate in the study. Cities eligible for entry into the study are: 1. Cities with at least four urban health centers and four health-care homes. Eligible entry centers: 1. Health homes with at least two "Behvarz". 2. City bases Have at least 2 health care staff. And preferably have recruiting staff . 3. health homes that are preferable to the Very small village .eligible staff : Preferably, have fixed forces (recruitment) of health centers and health homes At least 2 years into that center.
**Exclusion criteria:**
Disagreement with the text of the memorandum, by the relevant authorities of the universities

**Age**
From **30 years** old to **70 years** old

**Gender**
Both

**Phase**
N/A

**Groups that have been masked**
*No information*

**Sample size**
Target sample size: **32**
More than 1 sample in each individual
Number of samples in each individual: **40**
Thirty-two clusters are considered and for each cluster, 40 samples (individuals with inclusion criteria) will be provided.

**Randomization (investigator's opinion)**
Randomized

**Randomization description**
we use randomization to randomly assign centers for intervention groups

**Blinding (investigator's opinion)**
Not blinded

**Blinding description**

**Placebo**
Used

**Assignment**
Parallel

**Other design features**
This is a field trial. Interventions are performed at the first health level of the selected areas, and the final outcome is measured using population surveys.

## Secondary Ids

empty

## Ethics committees

**1**

**Ethics committee**
**Name of ethics committee**
national institute for medical research development
**Street address**
Tehran, West Fatemi St., Besat Street, No. 21
**City**
Tehran
**Province**
Tehran
**Postal code**
66900920-66938037

**Approval date**
2017-07-31, 1396/05/09

**Ethics committee reference number**
IR.NIMAD.REC.1396.084

## Health conditions studied

**1**

**Description of health condition studied**
Risk factors of Non-communicable diseases

**ICD-10 code**

**ICD-10 code description**

## Primary outcomes

**1**

**Description**
Population level of uncontrolled hypertension

**Timepoint**
At the beginning of intervention (Month 0), 12 months after starting of intervention, 24 month after starting of intervention

**Method of measurement**
Population Survey

**2**

**Description**
Population level of Poorly controlled diabetes
**Timepoint**
At the beginning of intervention (Month 0), 12 months after starting of intervention, 24 month after starting of intervention
**Method of measurement**
Population Survey

**3**

**Description**
Population level of insufficient physical activity
**Timepoint**
At the beginning of intervention (Month 0), 12 months after starting of intervention, 24 month after starting of intervention
**Method of measurement**
Population Survey

**4**

**Description**
Population level of current tobacco smoking
**Timepoint**
At the beginning of intervention (Month 0), 12 months after starting of intervention, 24 month after starting of intervention
**Method of measurement**
Population Survey

**5**

**Description**
Population level of insufficient intake of fruits/vegetables
**Timepoint**
At the beginning of intervention (Month 0), 12 months after starting of intervention, 24 month after starting of intervention
**Method of measurement**
Population Survey

**6**

**Description**
Population level of body mass index
**Timepoint**
At the beginning of intervention (Month 0), 12 months after starting of intervention, 24 month after starting of intervention
**Method of measurement**
Population Survey

## Secondary outcomes

empty

## Intervention groups

**1**

**Description**
Group IV: Assessment of the main NCDs’ risk factors and setting time-bound targets, AND Finding and sharing evidence on effective/efficient interventions for controlling the risk factors AND Operational planning with contribution of local health authoriti
**Category**
Other

**2**

**Description**
Group I: Assessment of the main NCDs’ risk factors and setting time-bound targets.
**Category**
Other

**3**

**Description**
Group II: Assessment of the main NCDs’ risk factors and setting time-bound targets AND Finding and sharing evidence on effective/efficient interventions for controlling the risk factors
**Category**
Other

**4**

**Description**
Group III: Assessment of the main NCDs’ risk factors and setting time-bound targets AND Finding and sharing evidence on effective/efficient interventions for controlling the risk factors AND Operational planning with contribution of local health authorit
**Category**
Other

**5**

**Description**
Control group: A county (Garmsar County) consisting of 4 health houses and 4 health center will not receive any intervention, and only three survey will be conducted.
**Category**
Other

## Recruitment centers

**1**

**Recruitment center**
**Name of recruitment center**
Iran university of medical sciences
**Full name of responsible person**
Maziar Moradi-Lakeh
**Street address**
Shahriar Health Network
**City**
Shahriar
**Province**

Tehran
**Postal code**
1449614535
**Phone**
+98 21 8860 2225
**Email**
mazmoradi@gmail.com

**2**

**Recruitment center**
**Name of recruitment center**
Bushehr University of Medical Sciences
**Full name of responsible person**
Maziar Moradi-Lakeh
**Street address**
Sports ST, Bushehr
**City**
Borazjan
**Province**
Boushehr
**Postal code**
1449614535
**Phone**
+98 71 3252 2078
**Email**
mazmoradi@gmail.com

**3**

**Recruitment center**
**Name of recruitment center**
Semnan University of Medical Sciences
**Full name of responsible person**
Maziar Moradi-Lakeh
**Street address**
Basij Blvd
**City**
Damghan
**Province**
Semnan
**Postal code**
3519899951
**Phone**
+98 23 3344 1022
**Email**
mazmoradi@gmail.com

**4**

**Recruitment center**
**Name of recruitment center**
Semnan University of Medical Sciences
**Full name of responsible person**
Maziar Moradi-Lakeh
**Street address**
Shahid Rajaie Street
**City**
Garmsar
**Province**
Semnan
**Postal code**
3519899951
**Phone**
+98 23 3422 8830
**Email**
mazmoradi@gmail.com

## Sponsors / Funding sources

**1**

**Sponsor**
**Name of organization / entity**
National Institute for Medical Research Development (NIMAD)
**Full name of responsible person**
Dr. Sayena Rafizadeh - Project #958058
**Street address**
No 21, Besat St, West Fatemi Ave
**City**
Tehran
**Province**
Tehran
**Postal code**
1449614535
**Phone**
+98 21 8860 2225
**Email**
Mazmoradi@gmail.com
**Grant name**
**Grant code / Reference number**
**Is the source of funding the same sponsor organization/entity?**
Yes
**Title of funding source**
National Institute for Medical Research Development (NIMAD)
**Proportion provided by this source**
100
**Public or private sector**
Public
**Domestic or foreign origin**
Domestic
**Category of foreign source of funding**
*empty*
**Country of origin**
**Type of organization providing the funding**
Other

## Person responsible for general inquiries

**Contact**
**Name of organization / entity**
Preventive Medicine and Public Health Research Center
**Full name of responsible person**
Maziar Moradi-Lakeh
**Position**
Professor
**Latest degree**
Specialist
**Other areas of specialty/work**
Public Health/Community Medicine
**Street address**

IUMS, Crossroads of Hemmat-Chamran expressways, Tehran, Iran
**City**
Tehran
**Province**
Tehran
**Postal code**
1449614535
**Phone**
+98 21 8860 2225
**Fax**
**Email**
mazmoradi@yahoo.com
**Web page address**

## Person responsible for scientific inquiries

**Contact**
**Name of organization / entity**
Preventive Medicine and Public Health Research Center
**Full name of responsible person**
Dr. Maziar Moradi-Lakeh
**Position**
Professor
**Latest degree**
Specialist
**Other areas of specialty/work**
Public Health/Community Medicine
**Street address**
Iran University of Medical Sciences, Hemmat-Chamran crossroads
**City**
Tehran
**Province**
Tehran
**Postal code**
1449614535
**Phone**
+98 21 8860 2225
**Fax**
**Email**
Mazmoradi@yahoo.com
**Web page address**

## Person responsible for updating data

**Contact**
**Name of organization / entity**
Preventive Medicine and Public Health Research Center
**Full name of responsible person**
Maziar Moradi-Lakeh
**Position**
Professor
**Latest degree**
Specialist
**Other areas of specialty/work**
Public Health/Community Medicine
**Street address**
IUMS, Crossroads of Hemmat-Chamran expressway
**City**
Tehran
**Province**
Tehran
**Postal code**
1635883813
**Phone**
+98 21 8860 2225
**Fax**
**Email**
mazmoradi@yahoo.com
**Web page address**

## Sharing plan

**Deidentified Individual Participant Data Set (IPD)**
Yes - There is a plan to make this available
**Study Protocol**
Yes - There is a plan to make this available
**Statistical Analysis Plan**
Yes - There is a plan to make this available
**Informed Consent Form**
Yes - There is a plan to make this available
**Clinical Study Report**
Yes - There is a plan to make this available
**Analytic Code**
Yes - There is a plan to make this available
**Data Dictionary**
Yes - There is a plan to make this available
**Title and more details about the data/document**
Access to relevant files is possible one year after the publication, with correspondence email: mazmoradi@gmail.com.
**When the data will become available and for how long**
One year after publication
**To whom data/document is available**
Academic researchers
**Under which criteria data/document could be used**
There is no limitation to the analysis
**From where data/document is obtainable**
Contact with email: mazmoradi@gmail.com
**What processes are involved for a request to access data/document**
Study aims of the study, about 10 to 21 days.
**Comments**
