# Supplementary material for: The effect of results-based motivating system on metabolic risk factors of non-communicable diseases: A field trial study
Source: PLoS One. 2024 Oct 17;19(10):e0311507. doi: 10.1371/journal.pone.0311507 (PMC11486381; doi:10.1371/journal.pone.0311507)
Supplement: S5 File — (PDF) [file pone.0311507.s007.pdf]

| | | Original protocol | | | | Revised protocol | | |
|---|---|---|---|---|---|---|---|---|
| Number of Medical Sciences Universities | | 2 | | | | 3 | | |
| Number of district | Intervention | 2 | | | | 3 | | |
| | Non- intervention | 0 | | | | 1 | | |
| | Total | 2 | | | | 4 | | |
| Number of field in each district | | | Urban | Rural | Total | Urban | Rural | Total |
| | | Intervention I | 2 | 1 | 3 | 1 | 1 | 2 |
| | | Intervention II | 2 | 1 | 3 | 1 | 1 | 2 |
| | | Intervention III | 2 | 1 | 3 | 1 | 1 | 2 |
| | | Intervention IV | 2 | 1 | 3 | 1 | 1 | 2 |
| | | Non- intervention | 0 | 0 | 0 | 4 | 4 | 8 |
| | | Total (all districts) | 16 | 8 | 24 | 16 | 16 | 32 |
