# Supplementary material for: The effect of results-based motivating system on metabolic risk factors of non-communicable diseases: A field trial study
Source: PLoS One. 2024 Oct 17;19(10):e0311507. doi: 10.1371/journal.pone.0311507 (PMC11486381; doi:10.1371/journal.pone.0311507)
Supplement: S6 File — (PDF) [file pone.0311507.s008.pdf]

Table 4. The mean difference in NCDs metabolic risk factors' levels between two surveys

NCDs Risk Factors	Intervention Package	First Survey (n=1,114)		Second Survey (n=1,219)		Mean Difference (95% Confidence Interval)
		Mean	SD	Mean	SD	
Systolic Blood Pressure	A	121.77	18.94	123.02	16.90	1.25 (-2.15, 4.64)
	B	120.89	18.13	127.64	20.22	6.75 (3.04, 10.46)
	C	123.98	17.96	120.72	17.68	-3.26 (-6.65, 0.12)
	D	118.11	16.35	122.93	16.99	4.82 (1.66, 7.98)
	None	118.14	15.12	122.72	15.02	4.58 (2.20, 6.96)
Diastolic Blood Pressure	A	81.32	10.77	80.77	10.05	-0.54 (-2.52, 1.43)
	B	79.66	10.64	82.84	12.02	3.18 (0.99, 5.38)
	C	80.05	10.16	80.74	10.76	0.68 (-1.31, 2.68)
	D	78.41	10.82	80.31	10.02	1.90 (-0.07, 3.87)
	None	76.04	10.33	78.66	9.44	2.63 (1.07, 4.19)
Total Cholesterol	A	171.50	32.71	175.48	40.15	3.98 (-4.88, 12.85)
	B	180.66	36.72	176.52	38.28	-4.14 (-12.59, 4.31)
	C	178.01	35.96	171.09	34.46	-6.92 (-14.47, 0.63)
	D	174.62	35.13	177.16	39.87	2.54 (-6.07, 11.14)
	None	168.83	36.34	173.70	34.84	4.86 (-1.76, 11.49)
BMI	A	27.81	5.24	28.19	5.19	0.38 (-0.62, 1.38)
	B	28.15	5.84	28.03	6.15	-0.12 (-1.24, 1.00)
	C	28.42	6.74	27.54	5.17	-0.88 (-2.05, 0.30)
	D	28.04	4.75	27.56	4.73	-0.48 (-1.38, 0.42)
	None	27.80	5.89	27.66	4.84	-0.14 (-1.00, 0.72)

Table 5. Estimated effects of intervention packages on the levels of NCDs metabolic risk factors

NCDs Risk factors	Intervention Package	Unadjusted: The Effect, β, with (95% Confidence interval)	Unadjusted: p-value	Adjusted for Socioeconomic Factors: The Effect, β, with (95% Confidence interval)	Adjusted for Socioeconomic Factors: p-value
Systolic Blood Pressure	A	-2.60 (-9.42, 4.23)	0.44	-3.03 (-10.42, 4.35)	0.40
	B	2.75 (-4.51, 10.00)	0.45	1.45 (-6.28, 9.18)	0.71
	C	-5.77 (-13.15, 1.60)	0.12	-7.88 (-15.98, 0.23)	0.06
	D	1.40 (-7.18, 9.97)	0.74	-0.77 (-8.91, 7.37)	0.85
	None	Reference Group			
Diastolic Blood Pressure	A	-3.46 (-8.01, 1.09)	0.13	-2.61 (-7.74, 2.51)	0.31
	B	0.27 (-4.75, 5.30)	0.91	-0.35 (-5.69, 4.99)	0.89
	C	-1.85 (-8.12, 4.42)	0.55	-3.61 (-9.30, 2.08)	0.21
	D	-1.35 (-9.19, 6.49)	0.73	-2.63 (-9.01, 3.75)	0.41
	None	Reference Group			
Total Cholesterol	A	-2.85 (-18.53, 12.82)	0.71	-1.59 (-16.70, 13.53)	0.83
	B	-10.41 (-26.78, 5.96)	0.20	-9.31 (-26.05, 7.43)	0.27
	C	-8.33 (-23.66, 7.00)	0.28	-9.54 (-27.49, 8.41)	0.29
	D	-4.59 (-15.29, 6.10)	0.39	-4.62 (-16.44, 7.20)	0.43
	None	Reference Group			
BMI	A	0.58 (-0.95, 2.12)	0.44	0.63 (-0.91, 2.17)	0.41
	B	0.49 (-1.30, 2.29)	0.58	0.57 (-1.34, 2.49)	0.55
	C	-0.65 (-2.41, 1.12)	0.46	-1.26 (-2.95, 0.43)	0.14
	D	-0.25 (-1.96, 1.46)	0.77	-0.55 (-2.17, 1.06)	0.49
	None	Reference Group			
