# Supplementary material for: The effect of results-based motivating system on metabolic risk factors of non-communicable diseases: A field trial study
Source: PLoS One. 2024 Oct 17;19(10):e0311507. doi: 10.1371/journal.pone.0311507 (PMC11486381; doi:10.1371/journal.pone.0311507)
Supplement: Attachment — Submitted filename: Response to Reviewers.docx [file pone.0311507.s010.docx]

PONE-D-23-20016

The effect of results-based motivating system on metabolic risk factors of non-communicable diseases: a field trial study

PLOS ONE

Dear Dr. Moradi-Lakeh,

Thank you for submitting your manuscript to PLOS ONE. After careful consideration, we feel that it has merit but does not fully meet PLOS ONE’s publication criteria as it currently stands. Therefore, we invite you to submit a revised version of the manuscript that addresses the points raised during the review process.

Please note that we have only been able to secure a single reviewer to assess your manuscript. We are issuing a decision on your manuscript at this point to prevent further delays in the evaluation of your manuscript. Please be aware that the editor who handles your revised manuscript might find it necessary to invite additional reviewers to assess this work once the revised manuscript is submitted.

The reviewer raises a concern about the statistical model used. Could you please carefully revise the manuscript to address this issue?

Please submit your revised manuscript by Dec 25 2023 11:59PM. If you will need more time than this to complete your revisions, please reply to this message or contact the journal office at plosone@plos.org. When you're ready to submit your revision, log on to https://www.editorialmanager.com/pone/ and select the 'Submissions Needing Revision' folder to locate your manuscript file.

Please include the following items when submitting your revised manuscript:

A rebuttal letter that responds to each point raised by the academic editor and reviewer(s). You should upload this letter as a separate file labeled 'Response to Reviewers'.

A marked-up copy of your manuscript that highlights changes made to the original version. You should upload this as a separate file labeled 'Revised Manuscript with Track Changes'.

An unmarked version of your revised paper without tracked changes. You should upload this as a separate file labeled 'Manuscript'.

If you would like to make changes to your financial disclosure, please include your updated statement in your cover letter. Guidelines for resubmitting your figure files are available below the reviewer comments at the end of this letter.

If applicable, we recommend that you deposit your laboratory protocols in protocols.io to enhance the reproducibility of your results. Protocols.io assigns your protocol its own identifier (DOI) so that it can be cited independently in the future. For instructions see: https://journals.plos.org/plosone/s/submission-guidelines#loc-laboratory-protocols. Additionally, PLOS ONE offers an option for publishing peer-reviewed Lab Protocol articles, which describe protocols hosted on protocols.io. Read more information on sharing protocols at https://plos.org/protocols?utm_medium=editorial-email&utm_source=authorletters&utm_campaign=protocols.

We look forward to receiving your revised manuscript.

Kind regards,

Steve Zimmerman, PhD

Senior Editor, PLOS ONE

Journal Requirements:

When submitting your revision, we need you to address these additional requirements.

1. Please ensure that your manuscript meets PLOS ONE's style requirements, including those for file naming. The PLOS ONE style templates can be found at

https://journals.plos.org/plosone/s/file?id=wjVg/PLOSOne_formatting_sample_main_body.pdf and

https://journals.plos.org/plosone/s/file?id=ba62/PLOSOne_formatting_sample_title_authors_affiliations.pdf

Response 1: We addressed all the PLOS ONE style requirements.

2. Thank you for stating the following financial disclosure:

"Research reported in this publication was supported by Elite Researcher Grant Committee under award number 958058 from the National Institutes for Medical Research Development (NIMAD), Tehran, Iran. MML received the award."

Please state what role the funders took in the study. If the funders had no role, please state: "The funders had no role in study design, data collection and analysis, decision to publish, or preparation of the manuscript."

If this statement is not correct you must amend it as needed.

Please include this amended Role of Funder statement in your cover letter; we will change the online submission form on your behalf.

Response 2: The funders had no role in study design, data collection and analysis, decision to publish, or preparation of the manuscript

3. In your Data Availability statement, you have not specified where the minimal data set underlying the results described in your manuscript can be found. PLOS defines a study's minimal data set as the underlying data used to reach the conclusions drawn in the manuscript and any additional data required to replicate the reported study findings in their entirety. All PLOS journals require that the minimal data set be made fully available. For more information about our data policy, please see http://journals.plos.org/plosone/s/data-availability.

Upon re-submitting your revised manuscript, please upload your study’s minimal underlying data set as either Supporting Information files or to a stable, public repository and include the relevant URLs, DOIs, or accession numbers within your revised cover letter. For a list of acceptable repositories, please see http://journals.plos.org/plosone/s/data-availability#loc-recommended-repositories. Any potentially identifying patient information must be fully anonymized.

Important: If there are ethical or legal restrictions to sharing your data publicly, please explain these restrictions in detail. Please see our guidelines for more information on what we consider unacceptable restrictions to publicly sharing data: http://journals.plos.org/plosone/s/data-availability#loc-unacceptable-data-access-restrictions. Note that it is not acceptable for the authors to be the sole named individuals responsible for ensuring data access.

We will update your Data Availability statement to reflect the information you provide in your cover letter.

Response 3: The data supporting this study’s findings cannot be shared publicly because of ethical and legal restrictions. Data are available upon request from the Preventive Medicine and Public Health Research Center, Iran University of Medical Sciences or National Institute for Medical Research Development after appropriate protocol submission to the institution’s office of Human Research Ethics Committee (contact via research.ethics.iums@gmail.com or research@ethics.ac.ir) for researchers who meet the criteria for access to confidential data. Prof. Maziar Moradi-Lakeh is head of the team and is responsible for considering data requests.

4. We note that the original protocol that you have uploaded as a Supporting Information file contains an institutional logo. As this logo is likely copyrighted, we ask that you please remove it from this file and upload an updated version upon resubmission.

Response 4: The Medical Journal of the Islamic Republic of Iran (MJIRI) is an editorially independent peer-reviewed online open-access journal owned and published by Iran University of Medical Sciences (IUMS), and this institutional logo belongs to IUMS.

[Note: HTML markup is below. Please do not edit.]

Reviewers' comments:

Reviewer's Responses to Questions

Comments to the Author

1. Is the manuscript technically sound, and do the data support the conclusions?

The manuscript must describe a technically sound piece of scientific research with data that supports the conclusions. Experiments must have been conducted rigorously, with appropriate controls, replication, and sample sizes. The conclusions must be drawn appropriately based on the data presented.

Reviewer #1: Partly

2. Has the statistical analysis been performed appropriately and rigorously?

Reviewer #1: No

3. Have the authors made all data underlying the findings in their manuscript fully available?

The PLOS Data policy requires authors to make all data underlying the findings described in their manuscript fully available without restriction, with rare exception (please refer to the Data Availability Statement in the manuscript PDF file). The data should be provided as part of the manuscript or its supporting information, or deposited to a public repository. For example, in addition to summary statistics, the data points behind means, medians and variance measures should be available. If there are restrictions on publicly sharing data—e.g. participant privacy or use of data from a third party—those must be specified.

Reviewer #1: Yes

4. Is the manuscript presented in an intelligible fashion and written in standard English?

PLOS ONE does not copyedit accepted manuscripts, so the language in submitted articles must be clear, correct, and unambiguous. Any typographical or grammatical errors should be corrected at revision, so please note any specific errors here.

Reviewer #1: Yes

5. Review Comments to the Author

Please use the space provided to explain your answers to the questions above. You may also include additional comments for the author, including concerns about dual publication, research ethics, or publication ethics. (Please upload your review as an attachment if it exceeds 20,000 characters)

Reviewer #1: Recommedation

Major Revision Required

I have no problems with the objectives or design of this well conducted trial. However, the authors should consider whether a simpler presentation of their results would suffice.

The authors describe what is clearly a well-designed and conducted field trial and should be congratulated for this. However, I am not convinced that the statistical model of line 202 is entirely appropriate. Thus, for example, Table 3 (line 310) shows the hypertension outcome in which the successive differences quoted for packages None, A, B, C, and D are +3.76, −2.02, −4,12, 1.47, −3.43. These are suggestive that Package does not have a linear effect and should not be considered as an ordered categorical variable as is indicated by the β in the statistical model (lines 202 and 207) but rather treated with dummy variables. Also, in the model CHC is regarded as an ordered categorical variable (coefficient θ) whereas it too should be modelled with dummy variables. Although this may cause some problems as the CHCs are nested within their respective packages. However, I do not think the broad conclusions are likely to be seriously affected but rather suggest that summarising the results as in Table 3 for example, (rather than fitting regression models) may be sufficient for this paper.

Response: Thank you very much for your careful review of the article. The statistical model presented in line 202 was used to estimate the effect of the four interventional packages on NCD Metabolic risk factors (NCDs MetRFs) applied to different sets of community health centers (CHC). This is a standard difference-in-difference specification in which the timing of potential exposure to the intervention is captured by the dummy variable named “Post”, and living in the catchment area of intervention CHCs is captured by a set of dummy variables represented by the vector “Package”. The effects of interest, then, are measured by the coefficients of the interaction of these two variables (Package × Post).^[1]^

Although it is not immediately clear from the specification of the statistical model, we generated dummy variables to represent these packages:

- The first dummy variable took the value 1 for all individuals surveyed in the no intervention CHCs, 0 otherwise;
- The second dummy variable took the value 1 for all individuals surveyed in CHCs that received the intervention package A, 0 otherwise;
- The third dummy variable took the value 1 for all individuals surveyed in CHCs that received the intervention package B, 0 otherwise;
- The fourth dummy variable took the value 1 for all individuals surveyed in CHCs that received the intervention package C, 0 otherwise;
- The fifth dummy variable took the value 1 for all individuals surveyed in CHCs that received the intervention package D, 0 otherwise;

We did not include the first dummy variable in the statistical model to avoid multicollinearity. Therefore, we estimated four $\beta$s and four $\rho$s. Since $\rho$s represent the effects of interest (i.e., the association of exposure to a specific intervention package), we only reported them in Table 4.

In addition, we included a separate dummy variable for each CHC to adjust for the effect of non-observable time-invarying factors specific to a CHC.

In sum, we used dummy variables to indicate different intervention packages and CHCs in the statistical model, while it appears we included them linearly. We have now revised the statistical model to reflect the exact variable specifications. We have also provided more descriptions for the statistical model in the text.

6. PLOS authors have the option to publish the peer review history of their article (what does this mean?). If published, this will include your full peer review and any attached files.

If you choose “no”, your identity will remain anonymous but your review may still be made public.

Do you want your identity to be public for this peer review? For information about this choice, including consent withdrawal, please see our Privacy Policy.

Reviewer #1: No

[NOTE: If reviewer comments were submitted as an attachment file, they will be attached to this email and accessible via the submission site. Please log into your account, locate the manuscript record, and check for the action link "View Attachments". If this link does not appear, there are no attachment files.]

While revising your submission, please upload your figure files to the Preflight Analysis and Conversion Engine (PACE) digital diagnostic tool, https://pacev2.apexcovantage.com/. PACE helps ensure that figures meet PLOS requirements. To use PACE, you must first register as a user. Registration is free. Then, login and navigate to the UPLOAD tab, where you will find detailed instructions on how to use the tool. If you encounter any issues or have any questions when using PACE, please email PLOS at figures@plos.org. Please note that Supporting Information files do not need this step.

Response: The figures revised by PACE.

1. Chapter 5.2.1 of Mostly Harmless Econometrics by Angrist and Pischke (2008), published by Princeton University Press.
