# Supplementary material for: The effect of results-based motivating system on metabolic risk factors of non-communicable diseases: A field trial study
Source: PLoS One. 2024 Oct 17;19(10):e0311507. doi: 10.1371/journal.pone.0311507 (PMC11486381; doi:10.1371/journal.pone.0311507)
Supplement: Attachment — Submitted filename: Responses to Reviewers.docx [file pone.0311507.s011.docx]

| **Date:** | Mar 19 2024 09:31PM |
| --- | --- |
| **To:** | "Maziar Moradi-Lakeh" moradilakeh.m@iums.ac.ir |
| **From:** | "PLOS ONE" plosone@plos.org |
| **Subject:** | PLOS ONE Decision: Revision required [PONE-D-23-20016R1] |

PONE-D-23-20016R1

The effect of results-based motivating system on metabolic risk factors of non-communicable diseases: a field trial study

PLOS ONE

Dear Dr. Moradi-Lakeh,

Thank you for submitting your manuscript to PLOS ONE. After careful consideration, we feel that it has merit but does not fully meet PLOS ONE’s publication criteria as it currently stands. Therefore, we invite you to submit a revised version of the manuscript that addresses the points raised during the review process.

Please submit your revised manuscript by May 03 2024 11:59PM. If you will need more time than this to complete your revisions, please reply to this message or contact the journal office at [plosone@plos.org](mailto:plosone@plos.org). When you're ready to submit your revision, log on to <https://www.editorialmanager.com/pone/> and select the 'Submissions Needing Revision' folder to locate your manuscript file.

Please include the following items when submitting your revised manuscript:

- A rebuttal letter that responds to each point raised by the academic editor and reviewer(s). You should upload this letter as a separate file labeled 'Response to Reviewers'.
- A marked-up copy of your manuscript that highlights changes made to the original version. You should upload this as a separate file labeled 'Revised Manuscript with Track Changes'.
- An unmarked version of your revised paper without tracked changes. You should upload this as a separate file labeled 'Manuscript'.

If you would like to make changes to your financial disclosure, please include your updated statement in your cover letter. Guidelines for resubmitting your figure files are available below the reviewer comments at the end of this letter.

If applicable, we recommend that you deposit your laboratory protocols in protocols.io to enhance the reproducibility of your results. Protocols.io assigns your protocol its own identifier (DOI) so that it can be cited independently in the future. For instructions see: [https://journals.plos.org/plosone/s/submission-guidelines#loc-laboratory-protocols](https://nam11.safelinks.protection.outlook.com/?url=https%3A%2F%2Fjournals.plos.org%2Fplosone%2Fs%2Fsubmission-guidelines%23loc-laboratory-protocols&data=05%7C02%7Cseyed.karimi%40louisville.edu%7C6c64239f0115418cc38a08dc4a7b3aa7%7Cdd246e4a54344e158ae391ad9797b209%7C0%7C0%7C638467140340442286%7CUnknown%7CTWFpbGZsb3d8eyJWIjoiMC4wLjAwMDAiLCJQIjoiV2luMzIiLCJBTiI6Ik1haWwiLCJXVCI6Mn0%3D%7C0%7C%7C%7C&sdata=qV6B9nCR7E6Re6ec22%2FQ1WCHNOylgaiK7giG%2FIH8x78%3D&reserved=0). Additionally, PLOS ONE offers an option for publishing peer-reviewed Lab Protocol articles, which describe protocols hosted on protocols.io. Read more information on sharing protocols at [https://plos.org/protocols?utm_medium=editorial-email&utm_source=authorletters&utm_campaign=protocols](https://nam11.safelinks.protection.outlook.com/?url=https%3A%2F%2Fplos.org%2Fprotocols%3Futm_medium%3Deditorial-email%26utm_source%3Dauthorletters%26utm_campaign%3Dprotocols&data=05%7C02%7Cseyed.karimi%40louisville.edu%7C6c64239f0115418cc38a08dc4a7b3aa7%7Cdd246e4a54344e158ae391ad9797b209%7C0%7C0%7C638467140340453530%7CUnknown%7CTWFpbGZsb3d8eyJWIjoiMC4wLjAwMDAiLCJQIjoiV2luMzIiLCJBTiI6Ik1haWwiLCJXVCI6Mn0%3D%7C0%7C%7C%7C&sdata=0t0f2kMZnlwc9nwk8cjf8pwk%2B1JDn6KZz24LaH9bqwk%3D&reserved=0).

We look forward to receiving your revised manuscript.

Kind regards,

Thaís São-João
Academic Editor
PLOS ONE

Additional Editor Comments:

Dear Maziar Moradi-Lakeh,
I would like to express our appreciation for your contribution to our journal and for the effort you have put into this work.

After careful consideration by our editorial board and external reviewers, I regret to inform you that your manuscript requires major revisions before it can be considered for publication. While your study addresses an important topic and presents potentially valuable insights, there are several significant issues that need to be addressed to enhance the quality and rigor of the paper.

Specifically, the reviewers have highlighted the following concerns:

1. Clarity and Organization: The overall organization of the manuscript needs improvement. The flow of ideas is not consistently logical, and certain sections lack clarity, making it challenging for readers to follow the argumentation.

2. Methodological Rigor: There are concerns regarding the methodology employed in the study. Certain aspects of the experimental design or data analysis need further clarification to ensure the validity and reliability of the findings.

3. Literature Review: The literature review provided in the manuscript could be more comprehensive. It is essential to provide a thorough review of relevant prior research to contextualize your study and demonstrate its novelty and significance.

4. Discussion and Conclusions: The discussion section requires further elaboration and interpretation of the results. Additionally, the conclusions drawn should be more firmly grounded in the findings of the study and supported by evidence.

I understand that revising the manuscript may require significant time and effort on your part, but I assure you that addressing these concerns will greatly enhance the overall quality and impact of your work. Therefore, I would like to invite you to revise your manuscript in accordance with the reviewers' feedback.


Thank you once again for considering PLOS ONE for the publication of your work. We look forward to receiving your revised manuscript.

Sincerely,


[Note: HTML markup is below. Please do not edit.]

Reviewers' comments:

Reviewer's Responses to Questions

**Comments to the Author**

1. If the authors have adequately addressed your comments raised in a previous round of review and you feel that this manuscript is now acceptable for publication, you may indicate that here to bypass the “Comments to the Author” section, enter your conflict of interest statement in the “Confidential to Editor” section, and submit your "Accept" recommendation.

Reviewer #1: (No Response)

Reviewer #2: (No Response)

2. Is the manuscript technically sound, and do the data support the conclusions?

The manuscript must describe a technically sound piece of scientific research with data that supports the conclusions. Experiments must have been conducted rigorously, with appropriate controls, replication, and sample sizes. The conclusions must be drawn appropriately based on the data presented.

Reviewer #1: Yes

Reviewer #2: Partly

3. Has the statistical analysis been performed appropriately and rigorously?

Reviewer #1: Yes

Reviewer #2: No

4. Have the authors made all data underlying the findings in their manuscript fully available?

The [PLOS Data policy](https://nam11.safelinks.protection.outlook.com/?url=http%3A%2F%2Fwww.plosone.org%2Fstatic%2Fpolicies.action%23sharing&data=05%7C02%7Cseyed.karimi%40louisville.edu%7C6c64239f0115418cc38a08dc4a7b3aa7%7Cdd246e4a54344e158ae391ad9797b209%7C0%7C0%7C638467140340461901%7CUnknown%7CTWFpbGZsb3d8eyJWIjoiMC4wLjAwMDAiLCJQIjoiV2luMzIiLCJBTiI6Ik1haWwiLCJXVCI6Mn0%3D%7C0%7C%7C%7C&sdata=pYnk77TCpkseX3P6ei%2BBECS6dhjn38kULXUDndaEf0E%3D&reserved=0) requires authors to make all data underlying the findings described in their manuscript fully available without restriction, with rare exception (please refer to the Data Availability Statement in the manuscript PDF file). The data should be provided as part of the manuscript or its supporting information, or deposited to a public repository. For example, in addition to summary statistics, the data points behind means, medians and variance measures should be available. If there are restrictions on publicly sharing data—e.g. participant privacy or use of data from a third party—those must be specified.

Reviewer #1: Yes

Reviewer #2: Yes

5. Is the manuscript presented in an intelligible fashion and written in standard English?

PLOS ONE does not copyedit accepted manuscripts, so the language in submitted articles must be clear, correct, and unambiguous. Any typographical or grammatical errors should be corrected at revision, so please note any specific errors here.

Reviewer #1: Yes

Reviewer #2: Yes

6. Review Comments to the Author

Please use the space provided to explain your answers to the questions above. You may also include additional comments for the author, including concerns about dual publication, research ethics, or publication ethics. (Please upload your review as an attachment if it exceeds 20,000 characters)

Reviewer #1: Minor Revision

The authors have addressed my concern re the statistical model in the earlier version of this paper. The problem was that in the modelling ‘ordered categorical’ assumptions were made where the use of ‘dummy variables’ would have been more appropriate. However, if as seems to be the case here (see Page 1, line 35), when using a ‘dummy variable’ approach essentially gives the same conclusions as the ‘ordered categorical’ approach then to present the results with this (simpler) modelling approach seems sensible. In such circumstances, the authors should make it clear in their presentation what has been done. So, I suggest the authors revert back to their model as expressed in Line 202 of their original submission but add a sentence or two to explain that the ‘categorical’ approach leads to the same conclusions than the more complex ‘dummy' approach. I apologise to the authors for raising this technical point as it does not seem to affect their conclusions.

*Authors’ response:* Thank you very much for your careful attention. We have added a sentence that refers to this matter in the paragraph right after the statistical model is presented. Specifically, we wrote: “Although an ordered structure was considered when designing the intervention packages, we included the intervention package indicators as dummy variables, as the effects of the interventions might not necessarily be ordered.”

Also, we added following sentence to the discussion section, right before the limitations subsection: “Although the intervention packages were ordered from simple to complex, the observed effects did not necessarily change this way.”

A minor comment to add. Reduce p-values to 2 significant figures in Table 2 (and elsewhere). For example, ‘0.831 to 0.83’ and ‘0.968 to 0.99’. However, for p-values of less than 0.001 the notation p <0.001 is acceptable.

*Authors’ response:* It is important to preserve the consistency in using the decimal throughout the document. We have now used 2 decimals for all p-values as well. Thank you!

There appear to be some misprints in the table as a p-value given as ‘0.001>’ should be ‘<0.001’.

*Authors’ response:* Thank you for catching the errors. We corrected them.

Reviewer #2: The study presents a well-defined and appropriately conducted research problem, objective and methods.

However, the interventions carried out are not clearly presented (method section).

*Authors’ response:* Thank you very much for this helpful comment. We have now replaced Fig. 3 with a new figure and added details and graphical scheme for packages.

The results contain a dense arrangement of data that is also unclear. I suggest that the presentation of the results is simple and summarized. There is a lot of information and it confuses reading.

*Authors’ response:* We greatly appreciate your comment as clarity is of the most importance for a research document. In an attempt to address it, we have now moved Tables 5 and 6, which are related to linear or non-dichotomous outcomes, to the appendix (s6 file) and siginifcantly reduced their description to a short paragraph at the end of the results secton. The paragraph is: “Similar analyses with non-dichotomous outcome variables for the studied NCDs MetRFs, presented in the S6 file, showed no statistically significant effects of the interventions. Close-to-significant effect of intervention package C was measured only for systolic blood pressure and BMI, confirming the results for dichotomous outcome variables.”

I also believe that the statistical model presented is not the best. Why use a dummy model? The way the results were presented is very confusing. The first reviewer made this consideration, however in a new round the authors did not adjust the manuscript in order to make it simpler.

*Authors’ response:* We appreciate your concern and detailed attention to the methodology. Referring to this comment, we have now further described this matter in the paragraph right after the statistical model is presented. Specifically, we wrote: “Although an ordered structure was considered when designing the intervention packages, we included the intervention package indicators as dummy variables, as the effects of the interventions might not necessarily be ordered.” Also, we added following sentence to the discussion section, right before the limitations subsection: “Although the intervention packages were ordered from simple to complex, the observed effects did not necessarily change this way.”

Therefore, I recommend that the article be rejected and the authors prepare a new version for another magazine in order to provide clarity and lightness in the presentation of information.

7. PLOS authors have the option to publish the peer review history of their article ([what does this mean?](https://nam11.safelinks.protection.outlook.com/?url=https%3A%2F%2Fjournals.plos.org%2Fplosone%2Fs%2Feditorial-and-peer-review-process%23loc-peer-review-history&data=05%7C02%7Cseyed.karimi%40louisville.edu%7C6c64239f0115418cc38a08dc4a7b3aa7%7Cdd246e4a54344e158ae391ad9797b209%7C0%7C0%7C638467140340469342%7CUnknown%7CTWFpbGZsb3d8eyJWIjoiMC4wLjAwMDAiLCJQIjoiV2luMzIiLCJBTiI6Ik1haWwiLCJXVCI6Mn0%3D%7C0%7C%7C%7C&sdata=aUv5Xgy864pu7xoIUeR4jXHIIpfq3twqxDZDvPa3%2BxI%3D&reserved=0)). If published, this will include your full peer review and any attached files.


If you choose “no”, your identity will remain anonymous but your review may still be made public.


**Do you want your identity to be public for this peer review?** For information about this choice, including consent withdrawal, please see our [Privacy Policy](https://nam11.safelinks.protection.outlook.com/?url=https%3A%2F%2Fwww.plos.org%2Fprivacy-policy&data=05%7C02%7Cseyed.karimi%40louisville.edu%7C6c64239f0115418cc38a08dc4a7b3aa7%7Cdd246e4a54344e158ae391ad9797b209%7C0%7C0%7C638467140340476589%7CUnknown%7CTWFpbGZsb3d8eyJWIjoiMC4wLjAwMDAiLCJQIjoiV2luMzIiLCJBTiI6Ik1haWwiLCJXVCI6Mn0%3D%7C0%7C%7C%7C&sdata=g3b0CcjWvMM%2BG5suWq7DivZfwKODOSPp2aXmRvqeyA4%3D&reserved=0).

Reviewer #1: No

Reviewer #2: No

[NOTE: If reviewer comments were submitted as an attachment file, they will be attached to this email and accessible via the submission site. Please log into your account, locate the manuscript record, and check for the action link "View Attachments". If this link does not appear, there are no attachment files.]

While revising your submission, please upload your figure files to the Preflight Analysis and Conversion Engine (PACE) digital diagnostic tool, [https://pacev2.apexcovantage.com/](https://nam11.safelinks.protection.outlook.com/?url=https%3A%2F%2Fpacev2.apexcovantage.com%2F&data=05%7C02%7Cseyed.karimi%40louisville.edu%7C6c64239f0115418cc38a08dc4a7b3aa7%7Cdd246e4a54344e158ae391ad9797b209%7C0%7C0%7C638467140340482799%7CUnknown%7CTWFpbGZsb3d8eyJWIjoiMC4wLjAwMDAiLCJQIjoiV2luMzIiLCJBTiI6Ik1haWwiLCJXVCI6Mn0%3D%7C0%7C%7C%7C&sdata=%2BiaDiiT31vQ8MOtEF76XVnhPJNM68wRWFrmI1FpHO4w%3D&reserved=0). PACE helps ensure that figures meet PLOS requirements. To use PACE, you must first register as a user. Registration is free. Then, login and navigate to the UPLOAD tab, where you will find detailed instructions on how to use the tool. If you encounter any issues or have any questions when using PACE, please email PLOS at [figures@plos.org](mailto:figures@plos.org). Please note that Supporting Information files do not need this step.

*In compliance with data protection regulations, you may request that we remove your personal registration details at any time.* [*(Remove my information/details)*](https://nam11.safelinks.protection.outlook.com/?url=https%3A%2F%2Fwww.editorialmanager.com%2Fpone%2Flogin.asp%3Fa%3Dr&data=05%7C02%7Cseyed.karimi%40louisville.edu%7C6c64239f0115418cc38a08dc4a7b3aa7%7Cdd246e4a54344e158ae391ad9797b209%7C0%7C0%7C638467140340488494%7CUnknown%7CTWFpbGZsb3d8eyJWIjoiMC4wLjAwMDAiLCJQIjoiV2luMzIiLCJBTiI6Ik1haWwiLCJXVCI6Mn0%3D%7C0%7C%7C%7C&sdata=Lq9BR8XcELU3jZFD7KeqdMjJ0g9hehB71nsq5AJJgbk%3D&reserved=0)*. Please contact the publication office if you have any questions.*
